# The Role of Autophagy and Mitophagy in Bone Metabolic Disorders

**DOI:** 10.7150/ijbs.46627

**Published:** 2020-07-30

**Authors:** Shuai Wang, Zhantao Deng, Yuanchen Ma, Jiewen Jin, Fangjie Qi, Shuxian Li, Chang Liu, Feng-Juan Lyu, Qiujian Zheng

**Affiliations:** 1 Department of Orthopedics, Guangdong Provincial People's Hospital, Guangdong Academy of Medical Sciences, 510080, China.; 2 Department of Endocrinology, The First Affiliated Hospital of Sun Yat-sen University.; 3 South China University of Technology-The University of Western Australia Joint Center for Regenerative Medicine Research, School of Medicine, South China University of Technology, Guangzhou, 510006, China.

**Keywords:** autophagy, mitophagy, bone metabolic disorder, osteoblast, osteoclast

## Abstract

Bone metabolic disorders include osteolysis, osteoporosis, osteoarthritis and rheumatoid arthritis. Osteoblasts and osteoclasts are two major types of cells in bone constituting homeostasis. The imbalance between bone formation by osteoblasts and bone resorption by osteoclasts has been shown to have a direct contribution to the onset of these diseases. Recent evidence indicates that autophagy and mitophagy, the selective autophagy of mitochondria, may play a vital role in regulating the proliferation, differentiation and function of osteoblasts and osteoclasts. Several signaling pathways, including PINK1/Parkin, SIRT1, MAPK8/FOXO3, Beclin-1/BECN1, p62/SQSTM1, and mTOR pathways, have been implied in the regulation of autophagy and mitophagy in these cells. Here we review the current progress about the regulation of autophagy and mitophagy in osteoblasts and osteoclasts in these bone metabolic disorders, as well as the molecular signaling activated or deactivated during this process. Together, we hope to draw attention to the role of autophagy and mitophagy in bone metabolic disorders, and their potential as a new target for the treatment of bone metabolic diseases and the requirements of further mechanism studies.

## Introduction

Bone metabolic disorders mainly include osteolysis, osteoporosis, osteoarthritis (OA) and rheumatic arthritis (RA). Osteolysis is induced by imbalance between bone formation and bone resorption. Osteoporosis is a classic age-related bone disease characterized by low bone mineral density, deterioration of bone microstructure, and subsequent increase in bone fragility 1. Fragility fracture is the most serious complication of osteoporosis. OA is a degenerative disease of the synovial joints, which is characterized by synovium inflammation, progressive degradation of cartilage and the associated extracellular matrix, osteophyte formation and subchondral bone sclerosis 2. OA is the most common reason of synovial joint pain and dysfunction in the older adults 3. The disease progression of OA affects not only articular cartilage, but also the entire joint, including subchondral bone, articular capsule, synovial membrane and surrounding muscles. The risk factors of primary OA include gender, ageing and obesity 4. RA is a chronic inflammatory autoimmune disease. The pathological characteristics of RA include synovial hyperplasia, cartilage degradation, fibroblast-like synoviocytes infiltration into cartilage and bone surfaces, and subchondral bone erosion 5.

Total hip arthroplasty (THA) is a standard treatment of patients suffering from severe end-stage joint diseases such as OA, RA and severe trauma. THA significantly reduces pain, improves joint function and overall quality of life 6. One late complication of THA limiting the survival of the patients is aseptic osteolysis 7, which is the major cause of THA failure. Osteolysis is caused by the imbalance between bone formation by osteoblasts and bone resorption by osteoclasts. In fact, evidences suggest that various cell types including osteoblasts, osteoclasts, macrophages, fibroblasts and lymphocytes are involved in the pathogenesis of bone metabolic disorders 8. Immune cells, such as Th17 cells, B cells, macrophages, neutrophils, mast cells and fibroblast-like synoviocytes, are important for inducing and maintaining synovial inflammation in RA 9, 10. However, the underlying mechanism is not fully understood.

Autophagy is a highly conserved self-degradative and energy dynamic recycling process in the proliferation, differentiation and maturation of eukaryotic cells, which could provide energy and basic substances for cellular homeostasis and survival through degradation of cytoplasmic misfolded or aggregated proteins, damaged organelles or macromolecules 11, 12. In mammalian cells, there are three primary types of autophagy: microautophagy, macroautophagy and chaperone-mediated autophagy, which all eventually end in the delivery of cytoplasmic materials to the lysosome for degradation and recycling 13. Although autophagy was originally believed to be a strictly nonselective process which randomly engulfs cytoplasmic contents, it has now been clear that autophagy also acts in a selective process 14. Mitophagy, the mitochondrial selective autophagy, targets damaged mitochondria for degradation through receptor mediated mechanisms. Mitophagy is a normal physiological activity taking place under healthy conditions, whereas it can also be accelerated under pathological conditions 15, or specific physiological conditions to promote glycolysis in retinal development 16. Mitophagy is required for the differentiation of reticulocyte to mature erythrocytes in rats 17, and for T cell development in mice thymus to remove excess mitochondria 18. Recently, emerging evidence suggested that abnormal autophagy and mitophagy levels will break the balance of bone metabolism and play a key role in bone metabolism disorders 19, 20.

In this review, we expect to summarize the current knowledge on the role of autophagy and mitophagy in the bone metabolic disorders, aiming to explore whether there is a potential relationship between autophagy/mitophagy and bone metabolic disorders, which will pave the way for further studies in the future.

## Osteoblasts and osteoclasts are two major types of cells contributing to bone metabolism

**Figure 1:**
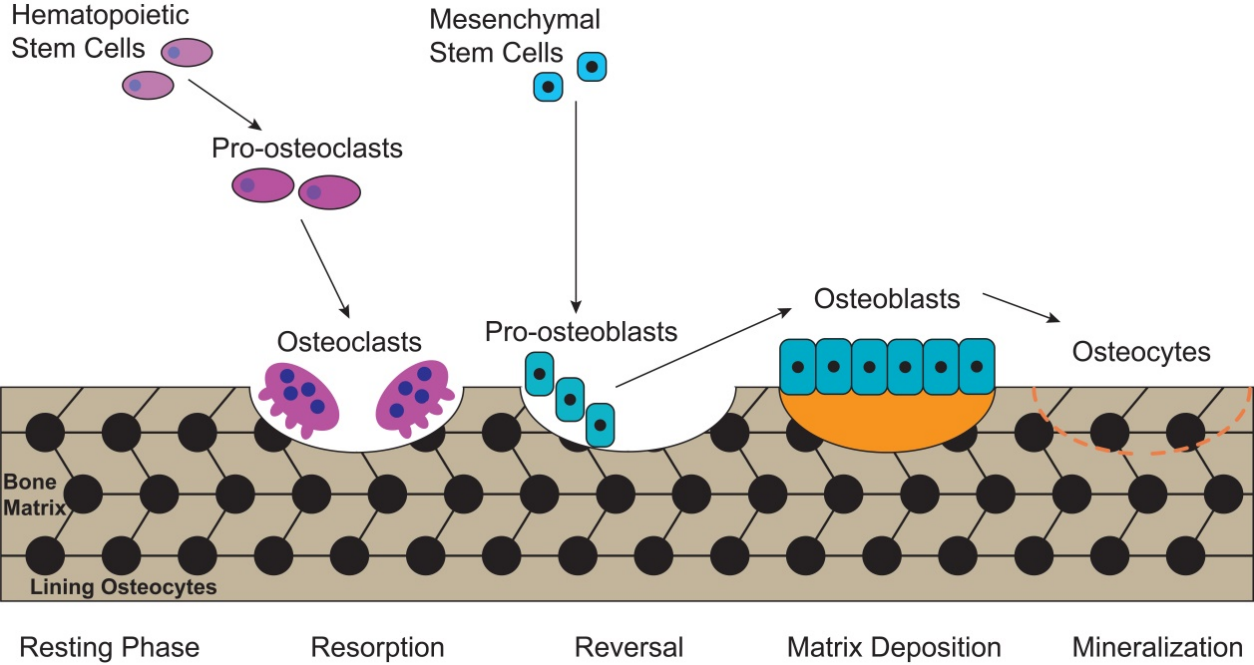
** The process of bone metabolic homeostasis.** Bone metabolic homeostasis is a continual process, including resorption, reversal, matrix deposition and mineralization. The osteoblasts which are derived from bone marrow mesenchymal stem cells, construct and shape the skeleton to get the maximal adaptability and terminally differentiate into osteocytes and form lining osteocytes. The osteoclasts, derived from hematopoietic stem cells, are multinucleated cells which can efficiently resorb bone.

Bone is a metabolically active tissue with network structure constituted by various types of cells and regulated by various factors. The maintenance of a stable bone metabolism requires the continuous differentiation and maturation of different kinds of stem cells, the mineralization of osteoblasts, the phagocytosis of osteoclasts and the secretion of a series of factors to regulate the interaction between cells. These require close coordination of intracellular organelles and regulatory factors, while consuming mass biological energy. Bone metabolic homeostasis is mainly maintained by a balance between bone formation by osteoblasts and bone resorption by osteoclasts, as illustrated in **Figure 1**. Different types of cells in the bone serve specific skeletal functions. Chondrocytes and osteoblasts, derived from bone marrow mesenchymal stem cells, construct and shape the skeleton to get the maximal adaptability. In contrast, the osteoclasts, which are derived from hematopoietic stem cells, can maintain mineral homeostasis by resorbing cancellous bone with a large surface area. Together, they play vital roles in the development and maintenance of bone size, shape and integrity 21.

There is a complicated inter-relationship between osteoblasts and osteoclasts. The activation and maturation of osteoclast precursors can be mediated by receptor activator of nuclear factor-κB ligand (RANKL) and intercellular adhesion molecule-1, which are expressed on the human cellular membrane of osteoblasts and bind with RANK and lymphocyte function-associated antigen 1 on the human cellular membrane of osteoclasts, respectively 22. Meanwhile, Tanaka et al. 22 proposed that intercellular adhesion molecule-1/lymphocyte function-associated antigen 1 adhesive pathway is a prerequisite for efficient interaction of RANKL with its receptor RANK. Moreover, Deng et al. 23 found that in the particle-induced osteolysis, wear particles could induce inflammatory responses in osteoblasts, with increased inflammatory cytokines at tissue level, including interleukin (IL)-6, IL-1β and tumor necrosis factor-α (TNF-α)^24^, leading to significantly increased number of activated osteoclasts and consequent local osteolysis. Meanwhile, osteoclasts can in turn affect the function of osteoblasts, which is directly regulated by the intercellular interaction or indirectly regulated by the expression of multiple cytokines such as semaphorin 4D 25.

## The definition of autophagy and mitophagy

The term “autophagy” is derived from the Greek meaning for “eating of self”. It was first presented by Deter et al. 26 over 50 years ago when they observed mitochondria and other intracellular structures in lysosome of rat liver cells treated by glucagon, a pancreatic hormone. Nowadays, we have more understanding of the process of autophagy at the molecular, cellular and tissue level 11, 12, 27, 28. A variety of autophagy related genes (*Atg*) have been revealed in yeast, plants, worms, flies and mammals, demonstrating that autophagy is a fundamental function in biological evolution. ATG proteins, produced by the transcription and translation of *Atg*, aggregate to form specific functional complexes which are activated and recruited to membranes to initiate autophagy. Autophagy begins with the formation of the 'autophagosome', an isolated double-membraned intermediate organelle (also termed phagophore), contained by some cytoplasmic materials such as soluble materials and organelles. Autophagosome matures through fusion with the lysosome, then become autolysosome and gains the ability to degrade the contents by lysosomal acid proteases 12.

**Figure 2:**
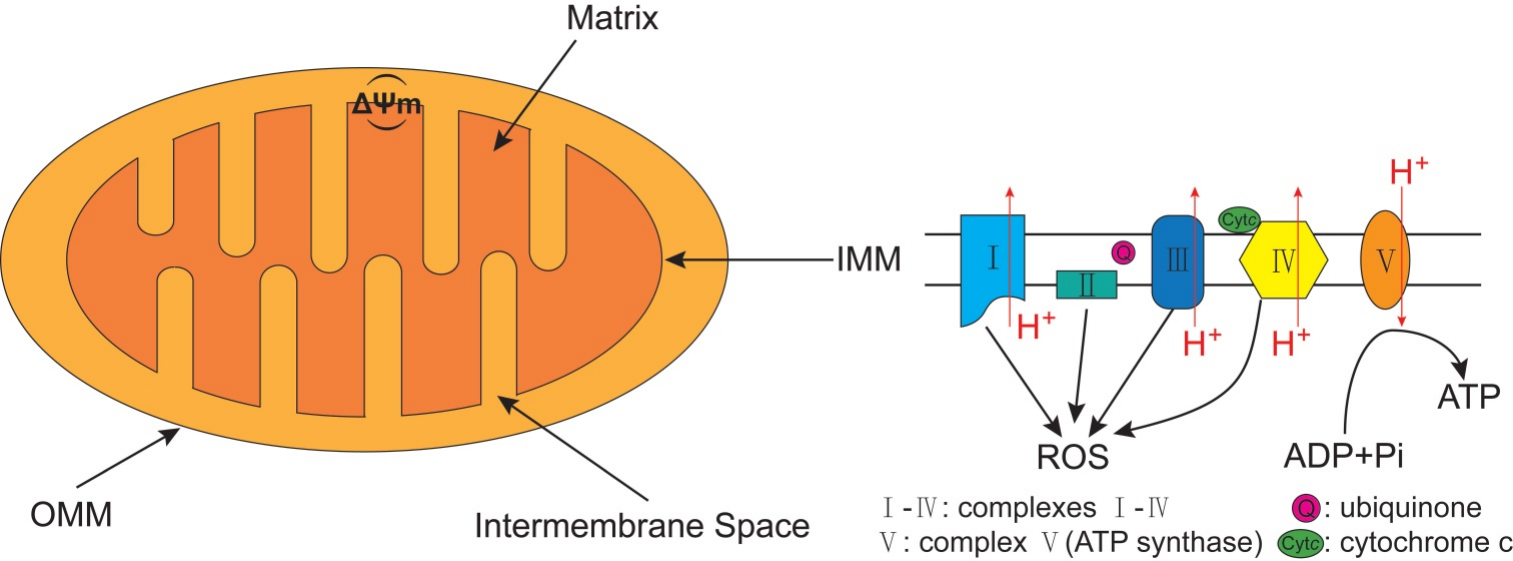
** The structure of mitochondrion and the electron transport chain (ETC).** The structure of mitochondrion includes a smooth outer mitochondrial membrane (OMM), a folded inner mitochondrial membrane (IMM), an intermembrane space and a central matrix. The ETC, including complexes I-IV, the electron transport ubiquinone and cytochrome c, is responsible for generating ATP and reactive oxygen species (ROS). The ETC produces the electrochemical potential energy contained in the proton (H^+^) gradient inside and outside of the IMM which can help to generate ATP from ADP and Pi by ATP synthase (complex V), as well as generate the mitochondrial membrane potential (ΔΨm).

Autophagy can be classified into non-selective autophagy and selective autophagy, with the former being a response to starvation, while the latter degrading damaged proteins and organelles selectively 29. 'Mitophagy', first proposed by Lemasters 30 in 2005, is the selective autophagy of mitochondria. Mitochondrion has cigar-shaped structure including a smooth outer mitochondrial membrane (OMM), a folded inner mitochondrial membrane (IMM), an intermembrane space and a central matrix (**Figure 2**) 31. The IMM accommodates the important components of the electron transport chain (ETC) and oxidative phosphorylation, which are responsible for generating adenosine triphosphate (ATP) and reactive oxygen species (ROS) 32. The mitochondrial ETC includes complexes I-IV, the electron transport ubiquinone and cytochrome c, meanwhile, the ETC produces the electrochemical potential energy contained in the proton (H+) gradient inside and outside of the IMM which can help to generate ATP from ADP and Pi by ATP synthase (complex V), as well as generate the mitochondrial membrane potential (ΔΨm) (**Figure 2**) 33, 34. Both the ΔΨm and ROS were increased when isolated mitochondria from rat heart was treated with H_2_O_2_ 35, while decreased ΔΨm usually lead to inhibit ROS production in mammalian cells 36. Furthermore, mitochondria play a crucial role in regulating intracellular calcium transport and ion levels by providing ATP for calcium transporting proteins and calcium signaling 37. Thus, the quantity and integrity of mitochondrial are important to maintain physiological function of cells. Mitochondrial dysfunction is represented by increasing ROS, depolarized ΔΨm, and increasing mitophagy. It is involved in various bone metabolic disorders, such as osteoporosis and OA 38-40. Therefore, restoring mitochondrial function by reducing the ROS content in mitochondria may be a potential therapeutic target for OA.

Mitophagy contributes to tissue homeostasis via reducing intracellular ROS produced by damaged mitochondria, recycling energy by limiting the energy requirements of invalid organelles, and producing ATP during the degradation under physiological and pathological conditions 41. In middle cortical artery occlusion-treated cerebral ischemia mice, acidic postconditioning-induced mitophagy mediated by Parkinson disease (autosomal recessive, juvenile) 2 (Parkin) can reduce neuronal injury caused by reperfusion following cerebral ischemia 42. However, the disordered mitophagy breaks mitochondrial homeostasis and impairs cellular energy metabolism and physiological function 41. In mouse cardiomyocytes, Overlapping with the m-AAA protease 1 homolog induced imbalance of mitochondrial fusion and fission events caused mitochondrial fragmentation mediated by optic atrophy protein 1, which facilitated removal of damaged mitochondria by mitophagy, and eventually triggered dilated cardiomyopathy and heart failure 43.

## The involvement of autophagy/mitophagy in osteoblasts and osteoclasts

Mitochondria play a vital role in the differentiation of bone precursor cells. During osteogenic differentiation of osteoblasts, a significantly increase in mitochondrial biogenesis, mitochondrial function (especially complex Ⅰ activity in the mitochondrial ECT) and ATP content were found 44. Mature osteoclasts exhibited increased mitochondrial protein content and lower intracellular ATP levels than those in osteoblasts and bone marrow macrophages 1, while the depletion of intracellular ATP resulted in distorted mitochondrial cristae in osteoclasts and stronger phagocytosis in osteoclasts, despite accelerated apoptosis of osteoclasts.

Mitophagy plays a key role in maintaining the homeostasis of healthy and damaged mitochondria in cells 45. The damaged mitochondria release ROS and apoptosis factors, which result in cell death or apoptosis by the activation of autophagy in osteoblasts. Impaired mitochondria can be degraded by mitophagy, which protect osteoblasts from apoptosis 46. Sun et al. found 17β-estradiol induced mitophagy in murine MC3T3-E1 osteoblast cell line and increased cell proliferation* in vitro*, highlighting the significance of estrogen for the clinical treatment of osteoporosis 47. In type 2 diabetes, loss of bone mass is a well-known risk factor correlated with osteoporotic fractures. Zhao et al. 48 found in type 2 diabetes related osteoporosis, osteoblasts had downregulated non-imprinted in Prader-Willi/Angelman syndrome region protein 2 (NIPA2), intracellular Mg^2+^ and activation of mitophagy, suggesting mitophagy negatively regulates osteogenesis. Yang et al. 49 used dexamethasone to treat rats with induced osteoporosis, and found that resveratrol further promoted SIRT1 (Sirtuin1)-induced mitophagy activation by suppressing phosphatidylinositol 3-kinase (PI3K)/protein kinase B (AKT)/mammalian target of rapamycin (mTOR) pathway. In addition, in inflammation-associated bone loss, such as bone resorption in apical periodontitis, simvastatin inhibited mitophagy and apoptosis of regional osteoblasts to modulate the balance between tissue destruction and regeneration 50.

Autophagy plays an important role in maintaining cellular function and homeostasis by recycling intracellular components. Piemontese et al. 51 deleted *Atg7* by an Osterix1-Cre transgene in mice to inhibit the autophagy level in osteoblasts, and found that inhibited autophagy leaded to the accumulation of endoplasmic reticulum and mitochondria and resulted in low bone mass and more fractures than the normal mice, it might be associated with decreased number of osteoblasts in the deleted *Atg7* mice. In type 2 diabetes, acceleration of autophagy in osteoblasts removed the increased fragmentation of mitochondria and swollen mitochondria to protect its ability of survive and differentiation, indicating that autophagy is important to maintain the survival and function of osteoblasts to limit increased ROS and protein oxidation caused by the high glucose environment 52.

## Signaling pathways involved in autophagy/mitophagy in osteoblasts and osteoclasts

Several signaling pathways have been demonstrated to involve in the regulation of autophagy/mitophagy in osteoblasts and osteoclasts. These signaling pathways as summarized in **Table 1.**

### The osteoblast and the pathway of the mitophagy: PINK1/Parkin

**Figure 3:**
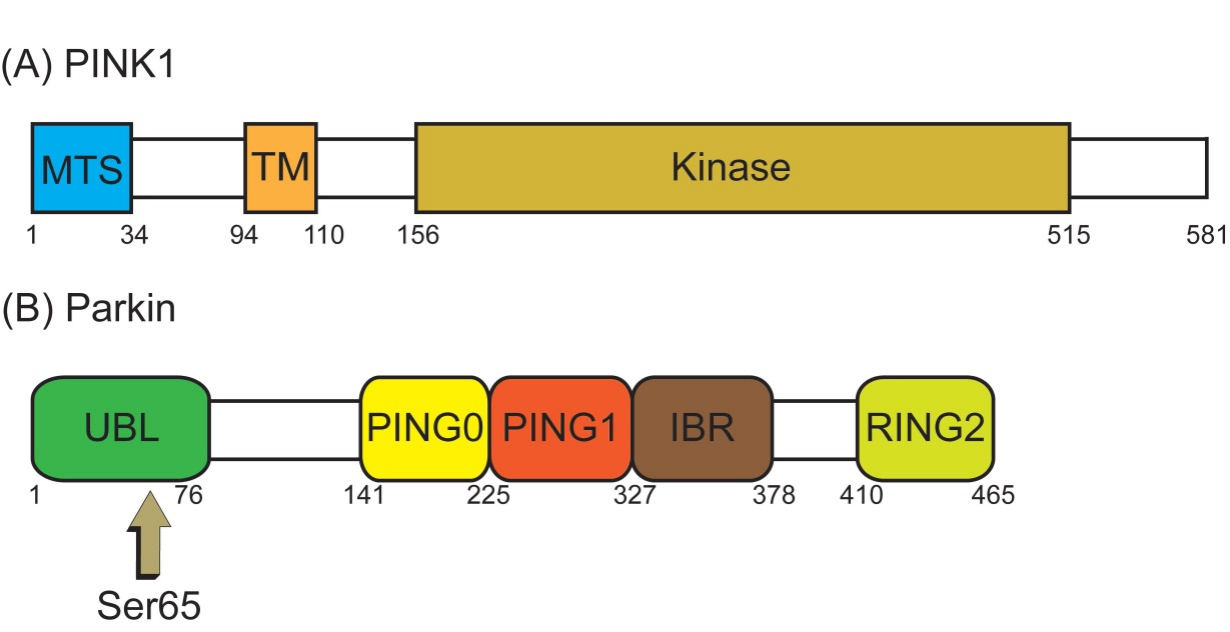
** The structure domain of PTEN-induced putative kinase 1 (PINK1) and Parkin.** (**A**) PINK1 possesses an N-terminal mitochondrial targeting sequence (MTS), an α-helical transmembrane (TM) segment, and a Ser/Thr kinase domain (Kinase). (**B**) Parkin contains an N-terminal ubiquitin-like (UBL) domain, three really interesting new gene (RING) domains (RING0, RING1, and RING2), and an in-between RING (IBR) domain between RING1 and RING2. PINK1 directly activates Parkin by phosphorylated Serine 65 (Ser65) in the UBL domain of Parkin.

The best known pathway of mitophagy is studied in mammalian cells and is mediated by PTEN-induced putative kinase 1 (PINK1) and Parkin 45. PINK1, a serine/threonine (Ser/Thr) kinase, is required in the Parkin mediated mitophagy 53, 54. PINK1 possesses an N-terminal mitochondrial targeting sequence (MTS), followed by an α-helical transmembrane (TM) segment, and a Ser/Thr kinase domain (**Figure 3A**). Parkin, an E3-ubiquitin ligase, is selectively recruited to dysfunctional mitochondria with low membrane potential by PINK1 55. The PINK1 and the E3-ubiquitin ligase Parkin are encoded by *PARK6* and* PARK2* genes, respectively 56. Parkin contains an N-terminal ubiquitin-like (UBL) domain, followed by three really interesting new gene (RING) domains, RING0, RING1, and RING2, and an in-between RING domain (IBR) between RING1 and RING2 (**Figure 3B**) 57. Although PINK1 is expressed in most types of tissue cells, the expression level is usually imperceptible under healthy conditions.

**Figure 4:**
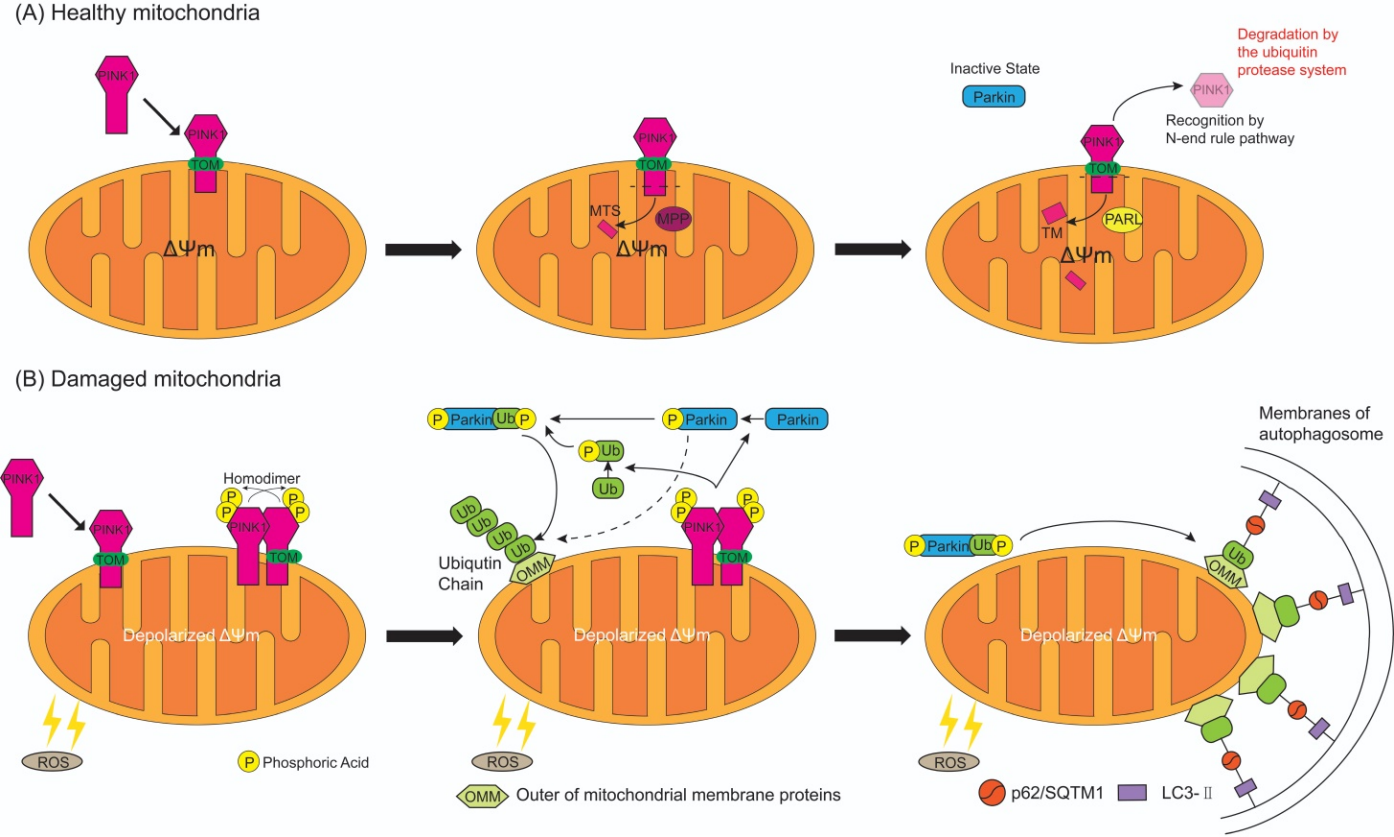
** The process of PINK1 and Parkin mediated mitophagy on healthy and damaged mitochondria.** (**A**) While PINK1 is recruited to the healthy mitochondria, N-terminal mitochondrial targeting sequence (MTS) of PINK1 is translocated across the mitochondrial membranes through the translocase of the outer membrane (TOM), and is exposed to central matrix depending on ΔΨm. Then MTS is cleaved by mitochondrial processing peptidase (MPP) in the mitochondrial matrix and the TM segment of PINK1 is cleaved by presenilin-associated rhomboid-like protease (PARL) in the inner membranes. The rest of PINK1 with an instable amino acid at the N-terminal is released to cytosol, recognized by N-end rule pathway and rapid degraded by the ubiquitin protease system. As a result, Parkin keep in an inactive state in the cytosol. (**B**) When the mitochondria are damaged by the reactive oxygen species (ROS), ΔΨm is depolarized and MTS cannot reach into the matrix which results in the escape of PINK1 from MPP/PARL-induced processing and N-end rule pathway-dependent degradation, therefore PINK1 steadily binds with the TOM. Two molecules of PINK1 form a homodimer and are intermolecularly phosphorylated to become highly active. Then PINK1 induces the phosphorylation of Parkin and ubiquitin at Ser65. Activated Parkin combine with or without phosphorylated ubiquitin can ubiquitinate many OMM proteins, but phospho-ubiquitin binded to Parkin can maximally activate E3-ubiquitin ligase activity of Parkin. In consequence ubiquitinated OMM proteins bind to the autophagosome by either direct binding to the LC3-II embedded in the membrane of autophagosome, or indirectly through p62/ Sequestosome 1 (SQSTM1), which contains a LC3 interacting domain and can bind to LC3, then promotes mitophagy.

In healthy circumstances (**Figure 4A**), while PINK1 is recruited to the healthy mitochondria, the N-terminal MTS of PINK1 is translocated across the OMM and IMM through the translocase of the outer membrane (TOM) and expose to central matrix with the aid of ΔΨm 57, and MTS is cleaved by mitochondrial processing peptidase (MPP) in the mitochondrial matrix, while the TM segment of PINK1 is cleaved by presenilin-associated rhomboid-like protease (PARL) in the inner membranes 58. This leaves an instable amino acid at the N-terminal of PINK1 which is then separated from the mitochondria to the cytosol for recognition by N-end rule pathway and rapid degradation by the ubiquitin protease system 59. Meanwhile, the UBL domain of Parkin negatively autoregulates its own E3-ubiquitin ligase activity at the N-terminus 60 to keep Parkin in an inactive state in the cytoplasm. Therefore, mitophagy is inhibited in normal cells with mitochondrial respiratory function.

Whereas, when the mitochondria are damaged by excessive ROS (**Figure 4B**), ΔΨm is depolarized which results in the escape of MTS/TM of PINK1 from MPP/PARL-induced processing and the rest of PINK1 escape of N-end rule pathway-dependent degradation. Instead, PINK1 steadily binds with the TOM 61, providing a platform for homodimer formation which is subsequent auto-phosphorylated. The resulting complex has a high kinase activity that promotes Parkin recruitment onto the damaged mitochondria 62. When PINK1 directly convert Parkin to an active state (formation of a ubiquitin-thioester intermediate) by phosphorylated Ser 65 in the UBL domain of Parkin (Figure 3B) 63, this posttranslational modification eliminates the autoinhibition of the UBL domain and induces Parkin activation. Activated Parkin ubiquitinates many OMM proteins, such as TOM20, mitofusins and voltage-dependent anion channel 1 64-66, for recruitment to phagophores that mature in to autophagosomes then fuse with lysosomes resulting in degradation of damaged mitochondria 67. Sufficient evidence indicates that ubiquitinated OMM proteins can bind to the phagophore by either direct binding to the autophagic adaptor protein light chain 3-II (LC3-II), which is embedded in the phagophore membrane 68, or through p62/Sequestosome 1 (SQSTM1), which contains a LC3 interacting domain to bind to LC3, then promote mitophagy 69. PINK1 can either regulate Parkin activation by another important substrate, ubiquitin 15, 57, 70. PINK1 mediates the phosphorylation at Ser65 of ubiquitin, then the phospho-ubiquitin Ser65 promotes activation of Parkin phosphorylated at Ser65 by PINK1, furthermore phosphorylation of both Parkin Ser65 and ubiquitin Ser65 greatly promote ubiquitin binds to Parkin to maximally activate E3-ubiquitin ligase activity of Parkin, thus ubiquitin is associated with efficient translocation of Parkin to damaged mitochondria (**Figure 4B**) 70-73. Evidence suggests that in addition to p62/SQSTM1, the E3-ubiquitin ligase Parkin stimulated ubiquitination of various OMM proteins 15 and mediated mitochondria as the cargo sequestrated into the autophagosomes by the Bcl-2/adenovirus E1B 19 kDa interacting protein 3 (BNIP3) binding with the LC3 and selective damaged mitochondria 74. The formed mitochondrial autophagosome will fuse with the lysosome to form the mitochondrial autolysosome resulting in degradation of the damaged mitochondria, thereby achieves quality control of mitochondria.

PINK1 and Parkin are the most extensively studied mitophagy-associated proteins. In the familial Parkinson's disease, loss-of-function mutations in Parkin and PINK1 caused accumulation of dysfunctional mitochondria and leaded to nigral neurodegeneration and early-onset Parkinson's disease, which indicates PINK1 and Parkin as key mediators of mitochondrial homeostasis and mitophagy 75, 76. The down-regulated expression of Parkin led to deteriorated osteogenic differentiation with dramatically reduced expression of bone morphogenetic protein 2 and collagen Ⅰ in adipose-derived mesenchymal stem cells (MSCs) 77.

### The osteoblast and the pathway of the mitophagy: SIRT1

The Sirtuins are a highly conservative protein family of nicotinamide adenine dinucleotide (NAD) dependent enzymes of deacetylases. They regulate the longevity of lower organisms, and regulate cellular and metabolic functions including stress resistance, genomic stability, tumorigenesis and energy metabolism, in mammals 78. Sirtuin 1 (SIRT1) was originally isolated in a screen for silencing factors and is the mammalian homolog of yeast silent information regulator 2 79. SIRT1 is an important regulatory factor of bone metabolism. Cohen-Kfir et al. 80 found that *Sirt1* knockout in female mice resulted in significant bone loss and decreased osteogenesis of MSCs and increased marrow adipogenesis. Sun et al. 81 attested that SIRT1 overexpression in MSCs repressed the acetylation of Forkhead box O3a (FOXO3a) and increased the expression of FOXO3a and superoxide dismutase 2 (SOD2), which promoted osteogenesis and reduced senility of MSCs. Meanwhile, wear particles could reduce the expression of SIRT1 in osteoblasts and osteolysis animal models 23. In contrast, the enhanced expression of SIRT1 in osteoblasts can dramatically reduce the expression level of particle-induced inflammatory cytokines and apoptosis of osteoblasts by NF-κB (nuclear factor-κB) and p53 signaling 23.

SIRT1 regulates the quality of mitochondria through mitophagy via different pathways in different cells. Deacetylation of the peroxisome proliferator-activated receptor gamma coactivator-1α (PGC-1α) mediated by SIRT1 plays a key role in mitochondrial metabolic control and mitochondrial biogenesis 82. However, SIRT1 is also reported to associate with mitochondrial degradation through mitophagy 83. Nicotinamide can enhance mitophagy and the effect is achieved through increasing the NAD^+^/NADH ratio and activating SIRT1 84. SIRT1 also regulates mitophagy by PINK1/Parkin pathway. A recent study on ischemic reperfusion in rat hearts demonstrated that resveratrol sequentially activated SIRT1, SIRT3, FOXO3a, PINK1, and PINK1 activated Parkin, leading to increased mitophagy 85. In the luminal epithelium of the human prostate cancer, loss of SIRT1 induced increased acetylation of SOD2 and reduced SOD2 activity, subsequently enhanced ROS production which induced the recruitment of Parkin to the mitochondria triggering mitophagy 86. In contrast, SIRT1 restoration could delay Parkin translocation to the mitochondria and reduce mitophagy 86.

### The osteoblast and the pathway of the autophagy: MAPK8/FOXO3

FOXO is a family of transcription factors playing a vital role in the cellular defense against oxidative stress. The FOXO family comprises four members: FOXO1, FOXO3, FOXO4 and FOXO6 87. Gomez-Puerto et al. 87 studied osteogenic differentiation of human MSCs and found that FOXO3 was activated by ROS, which was caused by increased mitochondrial metabolism for sufficient energy of differentiation, and the process was mainly dependent on mitogen-activated protein kinase 8 (MAPK8)-induced Ser294 phosphorylation of FOXO3, and FOXO3 activation is important in the control of ROS levels via the activation of autophagy. In fluoride-induced apoptosis of osteoblast, MAPK/JNK (Jun N-terminal kinase)-dependent autophagy acts as a protective role against apoptosis 88. Endoplasmic reticulum to nucleus signaling 1 (ERN1) can trigger autophagy, Wang et al. 19 observed that CoCrMo metal particles induced autophagy was mediated by MAPK8 after activated by ERN1.

### 4.2.1 The osteoclast and the pathway of the autophagy: Beclin-1/BECN1

Wear particles induced osteoclastogenesis is always an important cause of osteolysis in artificial joint replacement. Several molecules, including CD147, G-protein-coupled receptor kinase-interacting protein 1 (GIT1), IL-17A, kruppel-like factor 2 (KLF2), and tumor necrosis factor receptor-associated factor 6 (TRAF6) are reported to involve in the regulation of the autophagy in osteoclasts. Su et al. 89 found CD147 induced by wear particles activated autophagy, with increased level of Beclin-1 (Bcl-2 interacting coiled-coil protein) and soluble RANKL to promote osteoclastogenesis. Under starvation condition, GIT1 contributed to autophagy in osteoclasts through the disruption of the binding of Beclin1 with B-cell lymphoma-2 (Bcl2) by promoting the phosphorylation of Beclin1 at Thr119 90. IL-17A, an inflammatory cytokine, is also involved in RANKL-induced osteoclastogenesis by regulating the autophagic Beclin-1 activity 91. KLF2, a member of the zinc finger transcription factor family, which critically regulates embryonic lung development, and the function of endothelial cells, B-cells, T-cells and monocytes 92, could regulate autophagy in myeloid cells, which suppresses BECN1 expression and decreases histone H3K9 and H4K8 acetylation in the promoter region of *Becn1* during osteoclastogenesis 93. TRAF6, as an E3 ubiquitin ligase, mediated ubiquitination of Beclin1 at Lys117 for RANKL-stimulated osteoclast differentiation 94. Overall, Beclin-1/BECN1 pathway plays an important role in the autophagy of osteoclasts.

### The osteoclast and the pathway of the autophagy: p62/SQSTM1

SQSTM1/p62 is a characterized adaptor protein for autophagy in RANKL-induced osteoclastogenesis. During RANKL-induced osteoclast differentiation, the expression and localization of p62/SQSTM1 negatively correlated with LC3 accumulation and F-actin ring formation for regulating autophagic activation 95. Kaempferol, a flavonoid compound, can inhibit autophagy by degradation of p62/SQSTM1 and activate cell apoptosis during RANKL-induced osteoclastogenesis in murine macrophage (RAW264.7) cells 96. Paget's disease of bone is a bone disorder characterized by focal areas of abnormal, excessive bone turnover, specifically increased bone resorption and resulting in disorganized bone formation 97. In Paget's disease of bone, p62/SQSTM1 is a key regulator of ubiquitinated protein turnover through autophagy and ubiquitin-proteasome system, as well as positively stimulating NF-κB signaling and the oxidative stress-induced Kelch-like ECH-associated protein 1/NF-E2-related factor 2 (Keap1/Nrf2) pathway 98.

### The osteoclast and the pathway of the autophagy: mTOR

**Figure 5:**
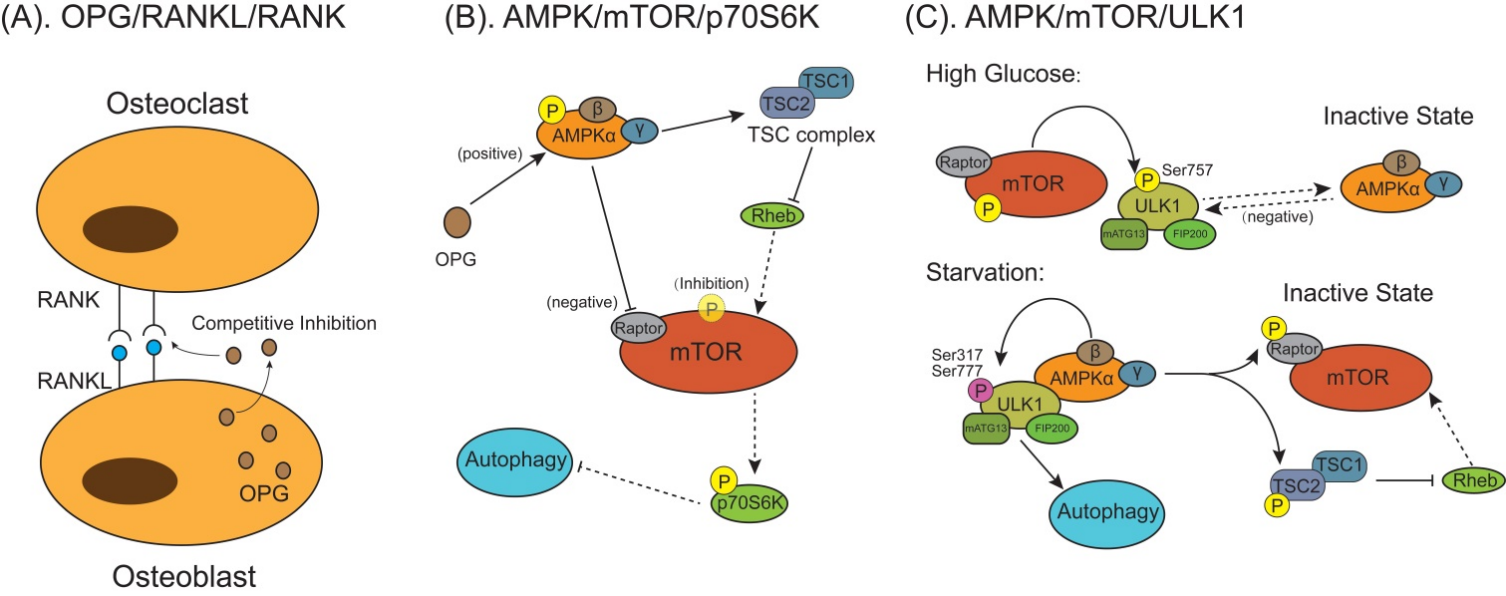
** The models for mammalian target of rapamycin (mTOR) behaviors on autophagic signaling pathway activation during osteoclast differentiation and formation.** (**A**) Osteoprotegerin (OPG) is a decoy receptor for the RANKL and competitively inhibit osteoclasts differentiation and maturation through blocking the interaction between RANKL and RANK. (**B**) OPG inhibits osteoclastogenesis and bone resorption by enhancing autophagy through activating AMPK/mTOR/p70S6K signaling pathway. OPG activates AMP‐activated protein kinase (AMPK) and downstream tuberous sclerosis complex 2 (TSC2). Activated AMPK inhibits mTOR through either phosphorylating Raptor on mTOR or promoting TSC1/TSC2 complex formation to inhibit Ras homolog enriched in brain (Rheb), which can induce mTOR activation. Reduction of activated mTOR activate autophagy indirectly by nonactivated 70‐kDa ribosomal protein S6 kinase (p70S6K). (**C**) The AMPK/mTOR/UNC-51 like autophagy activating kinase 1 (ULK1) signaling pathway mediated autophagy is involved in the regulation of energy metabolism in osteoclastogenesis. Under high glucose condition, activated mTOR phosphorylates ULK1 at Ser757, which inhibits the interaction between ULK1 and AMPK, to suppress autophagy. Under glucose starvation conditions when energy supply is exhausted, activated AMPK phosphorylates TSC2 and Raptor, which inhibits the activation of mTOR, then signal transduction from AMPK to ULK1 is restored. AMPK phosphorylates and activates ULK1 at multiple residues (Ser317 and Ser777), the activated ULK1/mATG13/FIP200 protein kinase complex lead to autophagy.

Mammalian target of rapamycin (mTOR) is one of several signal sensors to control the autophagic signaling pathway activation during osteoclast differentiation and formation (**Figure 5**). Osteoprotegerin (OPG) is a glycoprotein secreted by osteoblasts and is a key regulatory factor of bone activity. It is a decoy receptor for the RANKL with an equivalently high affinity as RANKL for its receptor RANK and can inhibit osteoclast differentiation and bone resorption by blocking the interaction between RANKL and RANK (**Figure 5A**) 99-102. Autophagy is found to participate in this process via AMP‐activated protein kinase (AMPK)/mTOR/70‐kDa ribosomal protein S6 kinase (p70S6K) signaling pathway (**Figure 5B**) 103. OPG enhanced the expression of p-AMPKα/AMPKα and downstream tuberous sclerosis complex 2 (TSC2). In the absence of energy or exogenous stimulus, AMPK can enhance TSC2 activation, promoting TSC1/TSC2 complex formation to inhibit Ras homolog enriched in brain (Rheb) activation 104. Tong et al. 103 found the OPG-induced increased TSC2 combined with TSC1 to form TSC complex, which deactivated the small G protein Rheb, thereby reducing mTOR activity. Owing to the activation of AMPK protein could inactivate mTOR activity through inhibiting Rheb, reduction of p-mTOR/mTOR expression activated autophagy accompanied by indirectly reducing p‐p70S6K/p70S6K expression 102. In other studies, suppression of the AMPK/mTOR/UNC-51 like autophagy activating kinase 1 (ULK1) signaling axis decreases autophagy in high-glucose-treated osteoclasts 105, which is attributed to that activated mTOR phosphorylates ULK1 at Ser757 under the high-glucose condition, which inhibits the interaction between ULK1 with its upstream activating factor AMPK then autophagy in response to energy stresses is suppressed (**Figure 5C**) 106. Under glucose starvation conditions when cellular energy is exhausted, AMPK is activated and phosphorylate two important mTOR factors, TSC2 and Raptor (**Figure 5C**) 107. In this condition, energy-consuming mTOR signaling pathway is turned off and activity is restrained, which allows signal transduction from AMPK to ULK1. AMPK phosphorylates and activates ULK1 at multiple residues (Ser317 and Ser777), forming activated ULK1/mATG13/focal adhesion kinase family interacting protein of 200 kDa (FIP200) protein kinase complex lead to autophagy 108-110.

### The osteoclast and the pathway of the autophagy: HIF-1α

Under hypoxic conditions, hypoxia-inducible factor-1α (HIF-1α) acts as a crucial role in the activation of autophagy. Zhao et al. 111 found that the up-regulated expression of HIF-1α-dependent Bcl-2/adenovirus E1B 19 kDa interacting protein 3 (BNIP3) was involved in hypoxic-induced activation of autophagy, which finally led to osteoclastogenesis. Under hypoxic conditions, in addition to acting as a protein signaling pathway to regulate autophagy, HIF-1α also mediated miRNAs which are involved in the regulation of autophagy in osteoclasts 112. During hypoxia induced osteoclast differentiation, up-regulated HIF-1α suppressed the expression of miRNA-20a on transcriptional level. However, miRNA-20a directly bound at the 3'-untranslated region of *Atg16l1* to negatively regulate ATG16L1 of autophagy, resulting in the regulatory axis of HIF-1α-miRNA-20a-*Atg16l1* activated autophagy in hypoxia induced osteoclast differentiation 112.

## The involvement of autophagy/mitophagy in bone metabolic disorders

### Osteolysis

Osteolysis is the major cause of THA failure with aseptic loosening as a severe complication. Its pathophysiology is not fully understood. The main pathogenic factor is prosthesis wear, resulting in wear particles around prosthesis and bone. In addition, other factors may contribute to the occurrence of osteolysis, such as the material and design of the prosthesis itself, the procedures performed during implantation, and a lack of connectivity at the interface between the implant and bone 113. Wear particles could provoke some pathologic conditions, such as inflammation, formation of foreign body granulomas and bone resorption by activated osteoclast, which ultimately lead to osteolysis 113. Current evidence suggests autophagy is an important factor in the pathogenesis of osteolysis 7. Recent evidence indicated that autophagy can be triggered by wear particles in the three main cell types involved in osteolysis, including osteoclasts, osteoblasts, and macrophages 19, 20, 24. Autophagy could induce the secretion of proinflammatory cytokines such as TNF-α, IL-6 and IL-8 or high mobility group box 1 in macrophages and osteoblasts, which have been implicated in the pathogenesis of aseptic loosening 114. Wear particles could stimulate the apoptosis of osteoblasts through enhancing their autophagy level, leading to osteolysis in a mouse calvarial resorption animal model 19. Wear alloy particles also induced differentiation of osteoclasts from bone marrow monocytes through stimulating autophagy in osteocytes which could reduce the expression of interferon-β 20. However, Li et al. 115 found that nano-sized Al2O3 particle induced autophagy in fibroblasts which reduced RANKL expression and negatively regulated the differentiation and maturation of osteoclasts, hence alleviated osteolysis. Furthermore, a few investigations of autophagy involvement in osteolysis in animal models indicated that the inhibition of autophagy decreased osteolysis severity 19, 24, 50, 51, 115. Therefore, inducing autophagy in fibroblasts to limit the activation of RANKL on osteoclasts may be a potential therapeutic target for the prevention and treatment of osteolysis.

### Osteoporosis

The primary osteoporosis includes postmenopausal osteoporosis and senile osteoporosis. 17β-estradiol deficiency is one of the main causes of postmenopausal osteoporosis, which can be treated by 17β-estradiol to protect osteoblasts by inducing mitophagy via the G protein-coupled receptor 30-extracellular regulated protein kinases 1/2 (ERK1/2) signaling pathway, however the protective effect could be abolished when the cells were pretreated with G15, a selective G protein-coupled receptor 30 antagonist 116. In addition to the signaling pathways involved in the imbalance between bone formation and resorption in osteoporosis, such as Wingless/Integrated (Wnt) pathway, Bone morphogenetic protein/Mothers against decapentaplegic homolog (BMP/Smad) pathway, RANKL/RANK pathway and TNF-α pathway 117, the mTOR pathway mediated autophagy (**Figure 5**) also regulates the regenerative function of MSCs to control the development of postmenopausal osteoporosis 118. Furthermore, oxidative stress, as a crucial primary factor for impaired osteoblastic bone formation in the osteoporosis, could be alleviated by early autophagy through endoplasmic reticulum stress pathway, including glucose-regulated protein 78 (GRP78) and protein kinase-like endoplasmic reticulum kinase (PERK) 119. SIRT1 is a key regulatory factor of mitophagy and can dramatically promote the autophagy level in osteoblasts to regulate bone metabolism. The drug candidates targeting SIRT1 may have a promising effect in treating osteoporosis and other bone metabolic disorders 120.

### OA

Autophagy plays an important role in the pathological development of OA. Chondrocyte apoptosis is involved in the pathogenesis of cartilage degeneration in OA. Chen et al. 121 found that conditioned medium of MSCs ameliorated the pathological development of OA in the rats by maintaining subchondral bone structure, producing more cartilage matrix, and inhibiting chondrocytes apoptosis with enhanced autophagy. Other evidences show that the enhancement of autophagic flux by isopsoralen could ameliorate IL-1β-stimulated apoptosis in rat chondrocytes 122. Furthermore, apoptosis of rat chondrocytes induced by hydrogen peroxide could be alleviated by global adiponectin-induced autophagy through the AMPK/mTOR pathway 123. Hydroxytyrosol (HT) has been used in the management of inflammatory diseases due to its anti-oxidative and anti-inflammatory pharmacological activities 124. Zhi et al. 125 found that HT could promote SIRT6-mediated autophagy, and control the progression of OA caused by inflammatory factors, such as IL-1β, IL‑6 and TNF-α. Meanwhile, Cetrullo et al. 126 also indicated that HT prevented oxidative stress-induced apoptosis of chondrocyte by inducing autophagy in a SIRT1-dependent as well as SIRT1-independent manners. More recently, transcription factor EB (TFEB) is identified as an important regulator of the autophagic flux by inducing lysosome biogenesis and promoting the autophagosome formation and fusion with lysosome 127. Accumulating evidence suggests that TFEB overexpression could ameliorate cartilage degradation post-surgery, through restraining the apoptosis and senescence of chondrocyte, and enhancing the autophagic flux 128. These researches suggest that autophagy plays a key role in the pathological development of OA.

The inflammatory process in OA also involves in the regulation of autophagy. As an important inflammation factor, IL-1β can induce experimental OA in chondrocytes 129. Inflammation reduced the autophagy level in rat chondrocytes 130, whilst activating autophagy attenuated the inflammation and the secretion of inflammatory factors in articular OA chondrocytes. In IL-1β stimulated OA chondrocytes, sucrose could activate autophagy through the activation of AKT/mTOR/P70S6K signaling pathway to block IL-1β-induced apoptosis and inflammatory reaction 131. Ansari et al. 132 indicated the extract of *Butea monosperma (Lam.)* flower has a strong ability to activate autophagy via suppression of mTOR pathway and suppressing IL-1β induced the expression of IL-6 and matrix-metalloproteases-3, -9 and -13 in human OA chondrocytes. Ozone treatment could suppress IL-1β induced IL-6 and TNF-α mRNA expression through promoting autophagy by activation of the AMPK/mTOR signaling pathway in OA chondrocytes 133. In addition, miRNA-335-5p alleviates OA chondrocytes inflammation by activation of autophagy to reduce the gene expression of IL-1β, IL-6 and TNF-α 134. Icariin protect against OA by suppressing inflammatory cytokines (IL-1, IL-6 and IL-12) and cells apoptosis, through activation of autophagy via NF-κB inhibition 135, Butein (2',3,4,4'-Tetrahydroxychalcone) activate autophagy through activating AMPK/TSC2/ULK1/mTOR pathway to inhibit IL-1β stimulated IL-6 expression in human chondrocytes with OA 136. All these evidences suggest that OA is associated with inflammation through suppressing autophagy.

**Figure 6:**
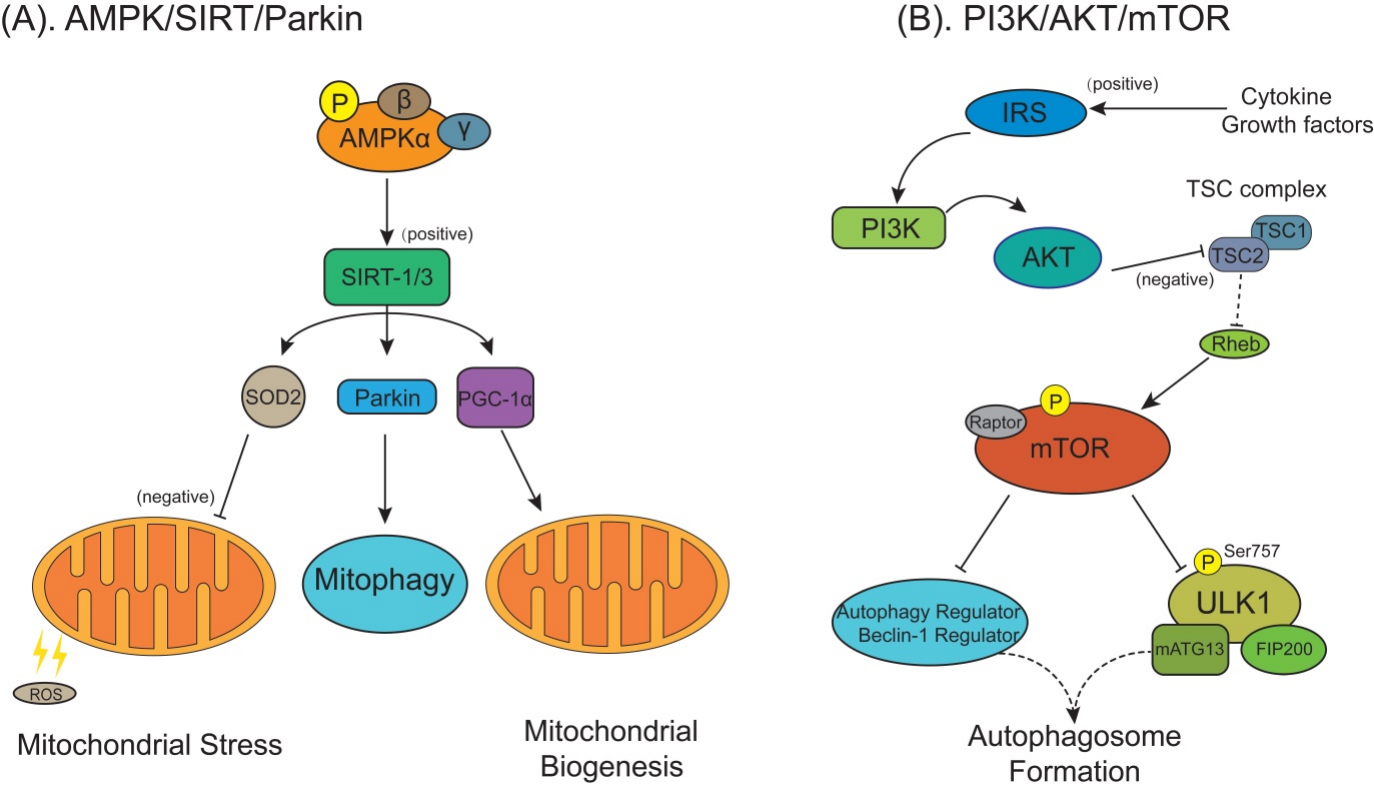
** Autophagy regulatory signaling pathways are involved in the pathological process of osteoarthritis/rheumatoid arthritis (OA/RA).** (**A**) The AMPK/SIRT signaling pathway in OA chondrocytes regulates the mitochondrial function, mitophagy and mitochondrial biogenesis and defenses against excessive ROS mediated by superoxide dismutase 2/Parkin/ peroxisome proliferator-activated receptor gamma coactivator-1α (SOD2/Parkin/PGC-1α). (**B**) The phosphatidylinositol 3-kinase/protein kinase B/mTOR (PI3K/AKT/mTOR) signaling pathway. Cytokines or growth factors (such as insulin) activate AKT signaling via PI3K through binging to insulin receptor substrate protein (IRS). Activated AKT inhibits TSC1/2 complex activity on Rheb, stimulating the activation of mTOR, which deactivates the ULK1/mATG13/FIP200 protein kinase complex, autophagy regulator and Beclin-1 regulator to prevent autophagosome formation.

In addition to autophagy, recent researches have also tried to explore the role of mitochondrial pathology in OA 137. Wang et al. 138 demonstrated that the prevention effect of metformin against OA was achieved through the upregulation of SIRT3-mediated PINK1/Parkin-dependent mitophagy. Huang et al. 139 found that in the monosodium iodoacetate-treated chondrocytes, which is a commonly used model for mimicking OA progression, zinc could reverse the negative effects of monosodium iodoacetate on energy metabolism of chondrocytes and eventually upregulate the PINK1-dependent selective mitophagy. In contrast, Shin et al. 140 indicated that in the chondrocytes derived from OA rat model and human primary OA patients, increased PINK1-mediated mitophagy contributed to degeneration of OA cartilage. Li et al. 141 indicated that plant homeodomain finger protein 23 (PHF23), a new autophagy inhibitor by promoting the degradation of E3 ligase, was overexpressed in human OA cartilage and synovium. Another study indicated that knockout of PHF23 prevent the chondrocytes apoptosis against IL-1β-induced OA by increasing autophagy, mitophagy, collagen II expression and reducing the OA-related proteins, which might make PHF23 a possible therapeutic target for OA 142. Fan et al. 143 demonstrated that 17β-estradiol, a steroid hormone, inhibited mitophagy by activating the PI3K/AKT/mTOR signaling pathway via the G-protein coupled estrogen receptor to exert protective effect on chondrocytes in OA. In addition, Blanco et al. 144 indicated their hypothetic view on the key role of AMPK-SIRT-Parkin in regulating mitochondrial function and defensing against excessive ROS in the chondrocytes of OA (**Figure 6A**).

### RA

RA is a chronic inflammatory condition 10, 145, during which osteoclasts differentiation is induced, and bone resorption is activated, mediated by the secretion of pro-inflammatory factors and RANKL in fibroblast-like synoviocytes. Evidence has suggested that autophagy is associated with the survival of fibroblast-like synoviocytes in RA 146. Autophagy also plays a key role in the maintenance of the central tolerance mechanism. The inhibition of autophagy blocks osteoclast differentiation from mouse macrophages and reduces bone erosion and the number of osteoclasts in a RA mouse model 147.

Similar to OA, autophagy is also involved in the mediation of the secretion and release of inflammatory cytokines in RA. In circulating immune cells, especially the CD4^+^ T and CD8^+^ T cells 148, increased LC3-II expression levels and decreased p62 expression levels indicated a higher level of autophagic flux in RA patients compared to healthy human 148. Lee et al. 149 found pretreatment of RA fibroblast-like synoviocytes with brazilin induced the activation of autophagy by inhibiting TNF-induced phosphorylation and degradation of IκBα (inhibitor of NF-κB) in these synoviocytes, and it could result in a significantly decrease of the secretion of TNF-induced proinflammatory cytokines IL-6 and IL-8. Liu et al. 150 found in the mice with collagen-induced arthritis, polypeptide composite selenium nanoparticles induced vascular endothelial cells to produce NO (nitric oxide), NO induced AMPK-α phosphorylation, which could inhibit mTOR phosphorylation, increase autophagy flux, inhibit NF-κB-p65 phosphorylation and reduce the level of inflammatory cytokines (IL-1β, IL-6 and TNF-α) in the polypeptide composite selenium nanoparticles treated mice RA immune cells. Zhou et al. 151 similarly found fumagillin prodrug nano-therapy induced endothelial NO production and activated AMPK, which subsequently modulated macrophage inflammatory response by inhibiting mTOR activity, increased autophagic flux, decreased degradation of IκB kinase, then suppressed the NF-κB-p65 signaling pathway and inflammatory cytokines release. Melatonin has beneficial effects on macrophage-associated diseases by regulating macrophage responses 152. Mitochondrial dynamics and mitophagy are shown to be involved in the regulatory functions of melatonin on macrophage polarization 152. Whether melatonin can regulate mitophagy to treat RA requires more studies.

Apoptosis defect is one of the causes of synovial hyperplasia in RA. Xu et al. 153 found a significant reverse correlation between apoptosis and autophagy in RA synovial tissues, which may be due to the deregulation of microRNA-30a targeting Beclin-1. Meanwhile, Fan et al. 154 also found that hypoxia could induce autophagy and elevate the expression of peptidyl arginine deiminase type IV (PADI4), as well as promoting the proliferation of fibroblast-like synoviocytes in a RA rat model. Consistently, the knockdown of *PADI4* was demonstrated to inhibit the hypoxia-induced autophagy and promote the apoptosis of fibroblast-like synoviocytes 154. Other evidence suggests that the rate of apoptosis in RA fibroblast-like synoviocytes is inversely related to the expression of Beclin-1 and LC3. Furthermore, the inhibition of protease system activation in fibroblast synovial cells resulted in increased LC3 expression and prolonged synovial cell lifespan 155. These studies suggested that increased autophagy in fibroblast-like synoviocytes contributes to synovial hyperplasia which promotes RA-associated synovitis.

In RA, altered autophagy levels are found not only in fibroblast-like synoviocytes but also in chondrocytes. The active participation of chondrocytes in local inflammation play a key role in RA, may cause a disruption of cartilage repair mechanisms resulting in cartilage destruction 156. Feng et al. found artesunate decreased PI3K/AKT/mTOR signals (**Figure 6B**) and increased B-cell lymphoma/leukemia-2-associated X protein (Bax), LC3-II/LC3-I and Beclin-1 in chondrocytes, resulting in inhibited chondrocyte proliferation and accelerated cell apoptosis and autophagy 157. It may indicate that treatment targeting chondrocytes in RA is a novel therapeutic strategy for the treatment of RA and the functional repair of bone inflammation.

## Conclusion and prospects

The osteoblasts and osteoclasts are important cellular components of bone. Osteoblasts, derived from bone marrow mesenchymal stem cells, are the crucial source of osteocytes, which represent the terminally differentiated state of the osteoblast lineage and the most abundant type of cells in bone. However, the osteoclasts, which derived from hematopoietic stem cells, are the only type of cell responsible for bone resorption. The balance between bone formation by osteoblasts and bone resorption by osteoclasts constitute the two major processes of bone remodeling. The level of autophagy and mitophagy could significantly affect the function and viability of osteoblasts and osteoclasts. Multiple factors are involved in regulating autophagy in cells, such as widely researched wear particles 113, hypoxic conditions 111, starvation conditions 105, even microgravity 158. Evidence suggested that wear particle induced osteoblasts apoptosis 19 and osteoclasts formation and differentiation through enhanced autophagy 89. These studies demonstrated that the inhibition of autophagy or mitophagy, by an autophagy inhibitor 3-methyladenine or siRNA knockdown of *Atg5,* could dramatically reduce the role of autophagy on function and proliferation of osteoblasts and osteoclasts *in vitro* and the severity of osteolysis *in vivo*. In contrast, though the role of mitophagy on bone cells is less studied, previous research has indicated that mitochondrial function and quantity are important to maintain osteoblasts and osteoclasts; therefore, more investigation would be desired to illustrate the involvement of mitophagy on bone metabolic disorders in the future. Up to date, no effective drug therapy to prevent or inhibit bone metabolic disorders has been confirmed. Surgical revision remains as the only treatment for terminal and severe bone metabolic disorders, such as aseptic loosening following THA. To date, in multitudinous signaling pathways involved in the autophagy and mitophagy, PINK1/Parkin and SIRT1 signaling pathways are the most extensively studied regulation factors of mitophagy, because of their crucial role in regulating mitophagy. In addition, other autophagy regulated factors, such as p62/SQSTM1, Beclin1, AMPK, mTOR1 and ATGs, form a complex and tightly connected regulatory network to mediate autophagy 157, 159. In this review, we summarize and discuss some signaling pathways that have been proved to have regulatory effects on autophagy and mitophagy in osteoblasts and osteoclasts during the physiopathologic change of bone metabolic disorders. Together, this evidence suggests a vital role of autophagy and mitophagy in bone metabolic disorders. Our previous study found that wear particles induced abnormal autophagy in osteoblasts and osteoclasts which could disrupt homeostasis of bone metabolism and lead to osteolysis, but the underlying mechanism remains unclear. Moreover, given the critical role of inflammatory cytokine secretion mediated by autophagy, inflammation could constitute a key factor in aseptic loosening pathology. This requires further study on the role of autophagy and mitophagy in bone metabolic disorders induced by stimulating factor like wear particles, to find specific regulated factor as a new underlying therapeutic target for preventing and controlling pathological process of osteolysis following THA.

## Figures and Tables

**Table 1:** Major autophagy/mitophagy regulation signaling pathways in the osteoblasts, MSCs and osteoclasts

Cells	Mechanisms
**Osteoblasts MSCs**	
PINK1/Parkin	1. PINK1 escapes from degradation and instead steadily binds with TOM, which recruits Parkin and Parkin ubiquitinates OMM proteins (TOM20, mitofusins, VDAC1), recognized by autophagy receptors, such as p62/SQSTM1 and LC3, then initiate mitophagy;
	2. PINK1/Parkin regulates the osteogenic differentiation of MSCs by PINK1/Parkin-induced mitophagy;
	3. In hypoxia-induced apoptosis of apical periodontitis, increased PINK1/Parkin was found which can up-regulate mitophagy activity.
SIRT1	1. SIRT1 directly participates in the regulation of metabolism and biogenesis of mitochondria;
	2. SIRT1 reduces the acetylation level of FOXO3a then increase the level of FOXO3a and SOD2 to achieve enhanced osteogenesis and reduced senescence of MSCs;
	3. SIRT1 dramatically reduces the expression of particle-induced inflammatory cytokines and the apoptosis of osteoblasts via NF-κB and p53 signaling.
MAPK8/FOXO3	1. FOXO3 is activated after Ser294 phosphorylation by MAPK8, then activated FOXO3 regulates ROS level in MSCs via the activation of autophagy;
	2. MAPK/JNK-dependent autophagy inhibits fluoride-induced apoptosis.
**Osteoclasts**	
Beclin-1/BENC1	1. Wear particles induce CD147 expression which can in turn induce Beclin-1 mediated autophagy to promote osteoclast formation;
	2. GIT1 promotes Bcelin-1 phosphorylation at Thr119 to disrupt the binding of Beclin-1 and Bcl2, and induce autophagy;
	3. KLF2 regulates autophagy by reducing BECN1 expression via decreased histone H3K9 and H4K8 acetylation in the promoter region of *Becn1* during osteoclastogenesis.
p62/SQSTM1	1. p62/SQSTM1 negatively correlates with LC3 accumulation and F-actin ring formation for regulating autophagic activation;
	2. p62/SQSTM1 is a key regulator of ubiquitinated protein turnover by affecting NF-κB signaling, as well as positively stimulating NF-κB signaling and the oxidative stress-induced Keap1/Nrf2 pathway;
mTOR	1. OPG enhances p-AMPKα and TSC2 expression. The activation of TSC2 by AMPK reduces mTOR activity via inhibiting Rheb activity, hence inhibits the mTOR mediated phosphorylation of p70S6K and reduces its inhibition on autophagy;
	2. Suppression of AMPK/mTOR/ULK1 signaling axis negatively regulates autophagy in diabetes;
	3. Activation of PI3K/AKT/mTOR pathway inhibits autophagy in hydrogen sulfide treated osteoclasts.
HIF-1α	1. Activation of HIF-1α-dependent BNIP3 promotes hypoxic-induced activation of autophagy;
	2. The regulatory axis of HIF-1α-miRNA-20a-*Atg16l1* activated autophagy in hypoxia induced osteoclast differentiation.

The process of autophagy/mitophagy is vital to proliferation and function of osteoblast, MSCs and osteoclast. The dysregulation of autophagy/mitophagy can lead to damaged mitochondria and accumulation of abnormal autophagy regulation factor, and induce apoptosis of osteoblasts or osteoclastogenesis in the bone metabolism disorders. The table lists the major regulated autophagy/mitophagy signaling pathways in the osteoblasts, MSCs and osteoclasts and possible potential pathogenic mechanisms. Abbreviations: AKT: protein kinase B; AMPK: AMP‐activated protein kinase;* Atg*: autophagy related genes; Bcl2: B-cell lymphoma-2; Beclin-1: Bcl-2 interacting coiled-coil protein; BNIP3: BCL2/adenovirus E1B 19 kDa protein-interacting protein 3; FOXO3a: Forkhead box O3a; GIT1: G-protein-coupled receptor kinase-interacting protein 1; HIF-1α: hypoxia-inducible factor-1α; JNK: Jun N-terminal kinase; Keap1: Kelch-like ECH-associated protein 1; KLF2: kruppel-like factor 2; LC3: light chain 3; MAPK8: mitogen-activated protein kinase 8; MSCs: mesenchymal stem cells; mTOR: mammalian target of rapamycin; NF-κB: nuclear factor-κB; Nrf2: NF-E2-related factor 2; OMM: outer mitochondrial membrane; OPG: Osteoprotegerin; p62/SQSTM1: Sequestosome 1; p70S6K: 70‐kDa ribosomal protein S6 kinase; Parkin: Parkinson disease (autosomal recessive, juvenile) 2; PI3K: phosphatidylinositol 3-kinase; PINK1: PTEN-induced putative kinase 1; Rheb: Ras homolog enriched in brain; ROS: reactive oxygen species; Ser: Serine; SIRT1: Sirtuin 1; SOD2: superoxide dismutase 2; TOM: translocase of the outer membrane; TSC2: tuberous sclerosis complex 2; ULK1: UNC-51 like autophagy activating kinase 1; VDAC1: voltage-dependent anion channel 1.

## Abbreviations

AMPK: AMP‐activated protein kinase
*Atg*: autophagy related genes
ATP: adenosine triphosphate
Beclin-1: Bcl-2 interacting coiled-coil protein
ETC: electron transport chain
FOXO: Forkhead box O
HIF-1α: hypoxia-inducible factor-1α
HT: hydroxytyrosol
IL: interleukin
IMM: inner mitochondrial membrane
LC3: light chain 3
MAPK: mitogen-activated protein kinase
MSCs: mesenchymal stem cells
mTOR: mammalian target of rapamycin
MTS: mitochondrial targeting sequence
NAD: nicotinamide adenine dinucleotide
OA: osteoarthritis
OMM: outer mitochondrial membrane
OPG: osteoprotegerin
SQSTM1: sequestosome 1
p70S6K: 70‐kDa ribosomal protein S6 kinase
Parkin: Parkinson disease (autosomal recessive, juvenile) 2
PINK1: PTEN-induced putative kinase 1
RA: rheumatic arthritis
RANKL: receptor activator of nuclear factor-κB ligand
Rheb: Ras homolog enriched in brain
RING: really interesting new gene
ROS: reactive oxygen species
Ser: serine
SIRT1: Sirtuin 1
SOD2: superoxide dismutase 2
THA: total hip arthroplasty
TM: transmembrane
TNF-α: tumor necrosis factor-α
TOM: translocase of the outer membrane
TSC: tuberous sclerosis complex
UBL: ubiquitin-like
ULK1: UNC-51 like autophagy activating kinase 1
ΔΨm: mitochondrial membrane potential
